# Suppressing a plant-parasitic nematode with fungivorous behavior by fungal transformation of a Bt *cry* gene

**DOI:** 10.1186/s12934-018-0960-5

**Published:** 2018-07-23

**Authors:** Chihang Cheng, Jialing Qin, Choufei Wu, Mengying Lei, Yongjun Wang, Liqin Zhang

**Affiliations:** 1 Collaborative Innovation Center of Zhejiang Green Pesticide, School of Forestry and Biotechnology, Zhejiang A&F University, Hangzhou, 311300 China; 2 School of Life Sciences, Huzhou University, Huzhou, 313000 China; 3 State Key Laboratory of Subtropical Silviculture, School of Forestry and Biotechnology, Zhejiang A&F University, Hangzhou, 311300 China; 4 Guangdong Eco-Engineering Polytechnic, Guangdong, 510520 China

**Keywords:** *Bacillus thuringiensis*, *Bursaphelenchus xylophilus*, ATMT, Fungus, Crystal protein, Nematicide

## Abstract

**Background:**

Pine wilt disease, caused by the pinewood nematode *Bursaphelenchus xylophilus* (PWN), is an important destructive disease of pine forests worldwide. In addition to behaving as a plant-parasitic nematode that feeds on epithelial cells of pines, this pest relies on fungal associates for completing its life cycle inside pine trees. Manipulating microbial symbionts to block pest transmission has exhibited an exciting prospect in recent years; however, transforming the fungal mutualists to toxin delivery agents for suppressing PWN growth has received little attention.

**Results:**

In the present study, a nematicidal gene *cry5Ba3*, originally from a soil *Bacillus thuringiensis* (Bt) strain, was codon-preferred as *cry5Ba3Φ* and integrated into the genome of a fungus eaten by PWN, *Botrytis cinerea*, using *Agrobacterium tumefaciens*-mediated transformation. Supplementing wild-type *B. cinerea* extract with that from the *cry5Ba3Φ* transformant significantly suppressed PWN growth; moreover, the nematodes lost fitness significantly when feeding on the mycelia of the *cry5Ba3Φ* transformant. N-terminal deletion of Cry5Ba3Φ protein weakened the nematicidal activity more dramatically than did the C-terminal deletion, indicating that domain I (endotoxin-N) plays a more important role in its nematicidal function than domain III (endotoxin-C), which is similar to certain insecticidal Cry proteins.

**Conclusions:**

Transformation of Bt nematicidal *cry* genes in fungi can alter the fungivorous performance of *B. xylophilus* and reduce nematode fitness. This finding provides a new prospect of developing strategies for breaking the life cycle of this pest in pines and controlling pine wilt disease.

**Electronic supplementary material:**

The online version of this article (10.1186/s12934-018-0960-5) contains supplementary material, which is available to authorized users.

## Background

Plant-parasitic nematodes (PPNs) are major pathogenic factors in many cash crops, including potato, soybean, and tomato [1, 2], and woody species, such as olive tree and pines [3, 4]. Destructive PPNs are very difficult to control because most of them are endoparasites. These nematodes spend most of their lives in the plant tissues, which can protect them from routine control strategies. Traditional control using highly toxic synthetic nematicides caused severe environmental problems and induced the production of chemical-resistant PPN strains. Biological agents for the control of PPNs have received greater attention in recent years, because they appear to be better solutions for crop protection against these devastating parasites. Bioactive compounds from plants and microorganisms [5, 6], novel nanoparticle delivery systems for biopesticides [7], nematophagous fungi and bacteria [8, 9], as well as advances in *in planta* transgenic or RNA interference technology [10–12] have significantly extended the outlook for controlling PPNs. However, relatively little attention has been paid to the microbial mutualists of nematodes that could be explored as promising targets for achieving efficient control.

The plant pathogenic pinewood nematode *Bursaphelenchus xylophilu*s (PWN) is in ecological balance with native pine species in North America [13], but it has become an invasive alien species in Japan, South Korea, and China, and spread into Portugal and Spain [4, 14]. This nematode is the major causal agent of pine wilt disease, which has devastated more than one million hectares of pine forests in China [15]. In recent years, compounds originally produced by *Streptomyces* species (such as avermectin, emamectin, milbemycin, and their derivatives) [16–18], nematicidal constituents screened from plants [19, 20], and endoparasitic fungi of nematode [21], were found applicable to be biopesticides for controlling PWN.

Crystal (Cry) proteins, produced by the soil bacterium *Bacillus thuringiensis* (Bt), are effective to control insects that destroy crops, as are the Cry proteins expressed in transgenic plants [22]. Moreover, increasing evidence has shown that Bt Cry proteins kill a wide range of nematodes [23, 24] and their nematicidal activities can be effectively delivered to crops for controlling PPNs through transgenic modification [25–27]. This suggested that using Bt *cry* gene-modified pine trees may be a sustainable and effective strategy for the conifer-parasitic nematode, PWN. Nevertheless, genetic engineering of tree genomes is highly challenging owing to the large costs and long-term evaluation of transgenic efficacy.

Although PWN mainly feeds on xylem parenchyma cells of pines during initial infection, the nematode eats blue stain fungi, which flourish later after the pine host is killed [28]. Spores of blue stain fungi could cling to the body surface of adult *Monochamus alternatus* and be transmitted to the twigs of healthy pine trees for the next cycle of PWN infection [29]. Ophiostomatoid fungi such as *Ophiostoma* and *Sporothrix* have been reported to be associated with PWN and the insect vector in different geographic regions [29–32]. PWN seems to benefit from the proliferation of blue stain fungi around the insect pupal chambers, because nematode reproduction increases when feeding on the blue stain fungi. Moreover, the abundance of mutualistic fungi correlates with the severity of pinewood disease [32]. The nutritional symbiotic partnership between PWN and its fungal associates implies that expressing toxic Bt protein from the fungus eaten by PWN might be a favorable alternative method for breaking the multispecies interactions among insect vectors, fungi, and the nematodes.

From the soil of the Tianmu Mountain, *Bacillus thuringiensis* zjfc85 was isolated and found to produce an approximately 130 kDa crystal protein Cry5Ba3, which acts as a strong nematicide against PWN, causing abnormal morphology within 48 h [33]. However, the characteristics of Cry5Ba3 and its potential applicability in biocontrol of PWN has not been well understood. In the present study, a codon-preferred *cry5Ba3Φ* gene was transformed into the filamentous fungus *Botrytis cinerea*, which is a good diet fungus for the laboratory population of PWN, via *Agrobacterium tumefaciens*-mediated transformation (ATMT). We demonstrate that fungal transformation with Bt expression provides significant nematicidal activity against PWN, suggesting a prospective strategy for delivering toxins by fungus to sites where the nematode forages. This method could be used to test the efficacies of the Cry protein family against parasitic nematodes with facultative fungivorous behavior.

## Methods

### PWN, *Botrytis cinerea*, and the *cry5Ba3* gene from *Bacillus thuringiensis* zjfc85

The PWN *Bursaphelenchus xylophilus* was first isolated from dead Masson pine trees in Zhejiang Province by Baermann funnels in 2010, and then cultured in the laboratory with the fungus *B. cinerea* (CGMCC NO.: 3.18906) on potato dextrose agar (PDA; BD Difco, Detroit, MI, USA).

*Bacillus thuringiensis* zjfc85 was one of 467 isolates in soils that were collected from Tianmu Mountain, Zhejiang Province [33]. Isolate zjfc85 exhibited significantly higher nematicidal activity than did the other collected strains, because of the single crystal protein Cry5Ba3 it harbors, which is approximately 130 kDa in mass (Additional file 1: Figure S1) [33].

### Construction of the plasmid pTFCM-*cry5Ba3Φ*

**Fig. 1:**
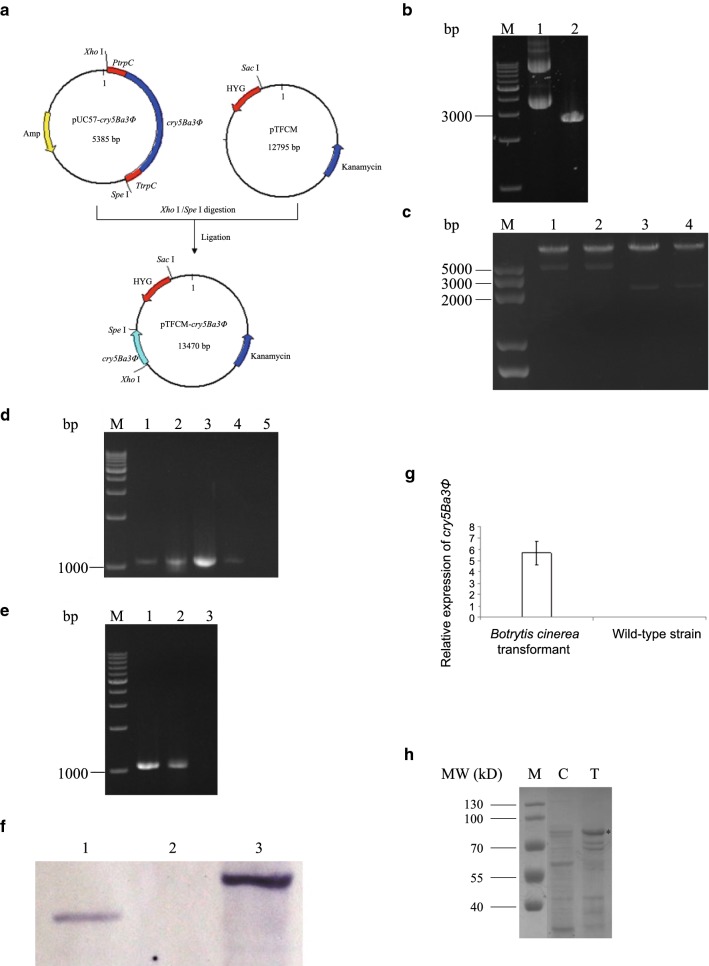
Recombination of *cry5Ba3Φ*-transgenic *Botrytis cinerea*. **a** Construction of the plasmid pTFCM-*cry5Ba3Φ*. HYG: hygromycin B resistant (hygromycin B phosphotransferase) gene; *PtrpC* and *TtrpC*: promoter and terminator of *Aspergillus nidulans*, respectively. **b** Plasmid pUC57-*cry5Ba3Φ* digested by *Spe*I and *Xho*I. M: DNA marker; 1: plasmid pUC57; 2: plasmid pUC57-*cry5Ba3Φ*. **c** Certification of the plasmid pTFCM-*cry5Ba3Φ*. M: DNA marker; 1–2: plasmid pTFCM-*cry5Ba3Φ* digested by *Sac*I and *Xho*I; 3–4: plasmid pTFCM-*cry5Ba3Φ* digested by *Spe*I and *Xho*I. **d** Identification of the AGL-1 pTFCM-*cry5Ba3Φ* by PCR amplification with cry-F/cry-R primers. M: DNA Marker; 1: plasmid pTFCM-*cry5Ba3Φ*; 2–4: AGL-1 pTFCM-*cry5Ba3Φ*; 5: AGL-1 pTFCM. **e** Southern blot analysis of genomic DNA of *cry5Ba3Φ*-transgenic *Botrytis cinerea*. DNA was digested with *Hin*dIII and probed with *cry5Ba3Φ*. 1: *cry5Ba3Φ*-transgenic *Botrytis cinerea*; 2: wild-type *Botrytis cinerea*; 3: plasmid pTFCM-*cry5Ba3Φ*. **f** Confirmation of *cry5Ba3Φ*-transgenic *Botrytis cinerea* by PCR amplification with cry-F/cry-R primers. M: DNA marker; 1: *cry5Ba3Φ*-transgenic *Botrytis cinerea*; 2: plasmid pTFCM-*cry5Ba3Φ*; 3: wild-type *Botrytis cinerea*. **g** Expression quantity of *cry5Ba3Φ* gene in *cry5Ba3Φ*-transgenic and wild-type *Botrytis cinerea* strains (*P* < 0.05). **h** SDS-PAGE analysis of soluble proteins produced by *cry5Ba3Φ*-transgenic *Botrytis cinerea*. An asterisk indicates the protein band of Cry5Ba3Φ protein. MW: protein molecular weight; M: pre-stained protein marker; C: *Botrytis cinerea* (pTFCM); T: *Botrytis cinerea* (pTFCM-*cry5Ba3Φ*)

According to the codon usage bias described previously [26], a new coding gene was designed to enhance Cry protein expression in filamentous fungi and named *cry5Ba3Φ* (GenBank Accession Number MG737676, Additional file 1: Figure S2), which encodes a protein consisting of 698 amino acids, with molecular mass approximately 78.5 kDa. The designed *cry5Ba3Φ* was then synthesized, combined with trpC promoter/terminator and sticky ends *Xho*I/*Spe*I, and linked with a pUC57 plasmid to obtain pUC57-*cry5Ba3Φ* (Genscript Co. Ltd., Nanjing, Jiangsu Province, China) (Fig. 1a). The plasmid pTFCM containing the T-DNA border repeat sequence and the *hph* gene, with *Aspergillus nidulans PtrpC*/*TtrpC* (Fig. 1a), was maintained in *Escherichia coli* DH5α and kept in National Joint Engineering Laboratory of Biopesticide Preparation, Zhejiang A&F University.

Plasmids (pUC57-*cry5Ba3Φ* and pTFCM) were extracted using Plasmid Mini Prepare (Axygen Biosciences, Union City, CA, USA) according to manufacturing instructions. These plasmids were then digested at the *Xho*I and *Spe*I sites, followed by dephosphorylation of the pTFCM plasmid and purifying the digested DNA segment containing *cry5Ba3Φ* by AxyPrep DNA gel recycle kit (Axygen Biosciences). The *cry5Ba3Φ* gene with *PtrpC* and *TtrpC* was ligated to the plasmid pTFCM by the action of T4 ligase at 16 °C overnight to obtain the plasmid pTFCM-*cry5Ba3Φ*. The ligated product pTFCM-*cry5Ba3Φ* was then determined by *Xho*I/*Spe*I and another set of restriction enzyme *Xho*I/*Sac*I.

### *Agrobacterium tumefaciens*-mediated transformation

*Agrobacterium tumefaciens* competent cells were prepared using 10% glycerol and the plasmid pTFCM-*cry5Ba3Φ* was transformed into *A. tumefaciens* AGL-1 strain according to an electroporation method [34]. Cells of *A*. *tumefaciens* AGL-1 strain (100 μl) carrying the plasmid pTFCM-*cry5Ba3Φ* were grown in 5 ml Luria–Bertani (LB) broth supplemented with 50 μg/ml kanamycin and streptomycin at 28 °C and 150 rpm for 12 h. After transferring 200 µl bacterial cells into 10 ml of induction medium (IM) [35] containing 200 µM acetosyringone (AS), they were grown for 6 h at 28 °C and 180 rpm. *A*. *tumefaciens* cells were finally diluted to achieve an optical density at 600 nm (OD_600_) of 0.15–0.3. The bacterial cells were mixed with an equal volume of *B. cinerea* (1 × 10^6^ conidia/ml); thereafter, 200 µl of the mixture was spread onto sterilized cellulose membranes (cellulose nitrate) with a pore size of 0.45 µm, overlaid on co-cultivation medium (IM + AS, 10 mmol/l glucose). After co-cultivation at 28 °C for 2 days, the membranes were transferred to PDA medium amended with 100 μg/ml hygromycin B and 50 μg/ml cefotaxime to select fungal transformants and to kill *A*. *tumefaciens* cells. After incubation at 28 °C for 3–5 days, individual colonies were cultured on potato dextrose broth (PDB) containing hygromycin B (100 μg/ml) and cefotaxime (50 μg/ml) at 28 °C for another 5 days.

### Genomic DNA isolation, polymerase chain reaction confirmation, and Southern blotting

Transformed and wild-type *B. cinerea* strains (100 μl of 1 × 10^6^ conidia/ml) were grown in 50 ml of PDB at 25 °C and 180 rpm for 7 days. The mycelia were filtered with autoclaved gauze, washed with sterile ddH_2_O three times, and then ground to powder in liquid nitrogen. Genomic DNA of *B. cinerea* was extracted using the Cetyltrimethylammonium Bromide (CTAB) method. Polymerase chain reaction (PCR) was used to confirm the presence of *cry5Ba3Φ* gene by amplifying a 1.2 kb region comprising portions of *hph* and *cry5Ba3Φ* genes. Primers cry-F (5ʹ-ACTACCTCAGACCACCACA-3ʹ) and cry-R (5ʹ-TCTCAAGCCTACAGGACAC-3ʹ) were internal in *cry5Ba3Φ* and *hph*, respectively. Each PCR consisted of 1 µg of genomic DNA, 2.5 U Taq DNA polymerase, 10 µl 10 × polymerase buffer, 1.5 µM MgCl_2_, 200 µM dNTP, and 0.5 µM each primer, adding ddH_2_O to adjust the volume of the reaction mixture to 50 µl. The cycling conditions were as follows: an initial denaturation of 5 min at 94 °C, followed by 35 cycles of 45 s for denaturation at 92 °C, 1 min for annealing at 58 °C, and 1 min for polymerization at 72 °C, with a final extension at 72 °C for 10 min.

Southern blotting was done to analyze the frequency of *cry5Ba3Φ* gene insertion in *B. cinerea*. Genomic DNA of the transformant and wild-type strains was extracted by DNeasy Mini Kit (Qiagen, Valencia, CA, USA). One hundred microgram of DNA from the strains and plasmid pTFCM-*cry5Ba3Φ* were digested with *Hin*dIII for 24 h, followed by electrophoresis on a 0.8% agarose gel at 1 V/cm for over 10 h in 0.5% Tris–acetate-ethylenediaminetetraacetic acid (EDTA) buffer. DNA fragments were transferred to a Nylon membrane using 10× SSC (saline sodium citrate; 1.5 M NaCl plus 0.15 M sodium citrate). The probe was prepared by PCR amplification using the primers of cry-Sf (5ʹ-TGCTGAAGCTGCTGTTC-3ʹ) and cry-Sr (5ʹ-CCTTGATGGTAGTTATGGGT-3ʹ) with the DIG-High prime DNA Labeling Kit I (Roche, Mannheim, Germany), according to the manufacturer’s instructions. Hybridizations were performed at 42 °C for 12–16 h and detected using the DIG-High Detection Kit I (Roche). After hybridization, the blots were washed twice in 2× SSC plus 0.1% sodium dodecyl sulfate (SDS) for 5 min at 25 °C. Thereafter, they were washed twice again in 0.5× SSC plus 0.1% SDS for 15 min at 42 °C.

### Real-time PCR analysis

Mycelia of the *B. cinerea* transformant and wild-type strains were collected, and the total RNA was extracted using AxyPrep™ multisource total RNA miniprep Kit (Axygen), followed by reverse transcription of mRNA with PrimeScript™ RT reagent Kit (TaKaRa, Tokyo, Japan). The PCR primers used were cry-rtF (5ʹ-CTCCCACTCACCCAACTC-3ʹ) and cry-rtR (5ʹ-TCACCCTTGGAAGCGTAT-3ʹ) and quantification of *cry5Ba3Φ* expression was performed using an Applied Biosystems 7300 Real-time PCR system (ABI, Foster City, CA, USA) with SYBR^®^ Premix Ex Taq™ II (TaKaRa). The housekeeping gene *β*-*actin* (GenBank Accession No. AJ000335) was chosen as control using the primers Bcactin-F (5ʹ-AAGTGTGATGTTGATGTCC-3ʹ) and Bcactin-R (5ʹ-CTGTTGGAAAGTAGACAAAG-3ʹ).

### Mitotic stability of *Botrytis cinerea* transformant

To determine the mitotic stability of the *B. cinerea* transformants with *cry5Ba3Φ*, they were cultured on PDA without hygromycin B for 5 days. After nine successive transfers, the colonies were tested for growth on PDA amended with 100 µg/ml hygromycin B. PCR amplification was further applied to confirm *cry5Ba3Φ* insertion using the primers cry-F and cry-R following the procedures mentioned above.

### Nematicidal activity of *Botrytis cinerea* transformant

*Botrytis cinerea* wild-type and transformant strains were cultured on PDB for 5 days at 25 °C and 180 rpm. Culture extracts from both the strains were filtered using 0.22 µm sterile filters. The solutions prepared for this test comprised 1 ml of extracts with different wild-type:transformant ratios: 1:0, 0.9:0.1, 0.5:0.5, 0.1:0.9, and 0:1. PWNs with mixed juvenile stages were washed out with sterile ddH_2_O from *B. cinerea* wild-type plates that have been fed by nematodes for 5 days. Thereafter, 50 µl of the suspensions (containing 600 nematodes on an average) were added into the extracts. Following 24 and 48 h of contact with extracts, live or dead nematodes were recorded. Five replicates were used in all the treatments.

In another experiment, 50 µl of the nematode suspensions were added to PDA freshly grown with wild-type and *cry5Ba3Φ*-transgenic *B. cinerea* strains, respectively. After 5 days, the nematodes were flushed with 3 ml sterile distilled H_2_O and the number of live or dead nematodes were recorded. Six replicates were used for each treatment.

### *Botrytis cinerea* transformants with different lengths of *cry5Ba3Φ*

National Center for Biotechnology Information (NCBI) Conserved Domain search (CD-search) analysis predicted that Cry5Ba3Φ contains an endotoxin-N (aa 91–327) and an endotoxin-C (aa 562–695). According to the structure of homologous crystal protein Cry5Ba (GenBank no. Q45712, Additional file 1: Figure S1), endotoxin-N of Cry5Ba3Φ may contain several helical regions (aa 115–161, 175–177, 187–207, 208–211, 222–256, 262–290, 299–315, 321–326) and endotoxin-C may consist of two helical regions (aa 571–573 and 584–586) and several beta-strand regions. Based on this information, aa 115, 202, 560, and 572 were selected to break down endotoxin-N and endotoxin-C, respectively. To determine whether integrity of segments anterior to endotoxin-N affects Cry5Ba3Φ nematicidal activity, another locus, aa 74, was selected.

Six pairs of primers were prepared to amplify fragments of *cry5Ba3Φ* aa 74–698 (1875 bp), 115–698 (1758 bp), 202–698 (1497 bp), 1–572 (1719 bp), 1–560 (1683 bp), and 74–572 (1503 bp). The pTFCM-TRP vector backbone was amplified using pTFCM-*cry5Ba3Φ* as template and pTFCM-phiF/-phiR as primers (pTFCM-phiF: 5ʹ-TACCTATTCTACCCAAGCATCCAAGATATCAGTAGATGCCGACCGGGA-3ʹ; pTFCM-phiR: 5ʹ-TTGGATGCTTGGGTAGAATAGGT-3ʹ). The steps mentioned above have been described in detail in Additional file 1: Methods. Vector (300 ng) and DNA fragment (1.20 µg) were ligated using GIBSON Assembly Cloning Kit (New England Biolabs, Ipswich, MA, USA). The ligated products were transformed into *E. coli* XL10-Gold for multiplication followed by *A. tumefaciens*-mediated transformation as described above, which resulted in the six *B. cinerea* transformant strains with different lengths of *cry5Ba3Φ* genes. During the above process, primers hph-F (5ʹ-TTCGATGTAGGAGGGCGTGGAT-3ʹ) and hph-R (5ʹ-CATTGCAGATGAGCTGTATCTGG-3ʹ) were used to determine whether truncated *cry5Ba3Φ* genes were successfully transformed into AGL-1.

### Confirmation of *Botrytis cinerea* with truncated *cry5Ba3Φ* by PCR and SDS-PAGE

Each of the six *B. cinerea* transformant strains were cultivated in 5 ml of PDB supplemented with the antibiotics hygromycin B and cefotaxime at 28 °C for 5 days. Genomic DNA was then extracted for PCR amplification using primers IDF (5ʹ-ACTAGTCATTGCAGATGAGCTG-3ʹ) and IDR (5ʹ-ACTAGTCATTGCAGATGAGCTGTATCTGGA-3ʹ) to certify the presence of truncated *cry5Ba3Φ* genes with different lengths. The PCR program has been presented in Additional file 1: Methods.

A colony of each of the successfully transformed *B. cinerea* strains was transferred to 50 ml of PDB containing hygromycin B and cefotaxime at 28 °C for 5 days. After centrifugation, the pellet of each strain was resuspended in 0.5% NaOH for repeated freeze–thaw cycles. Soluble proteins, including the Cry proteins, were detected by sodium dodecyl sulfate polyacrylamide gel electrophoresis (SDS-PAGE) followed by Coomassie blue staining.

### Nematicidal activity of *Botrytis cinerea* transformants with truncated *cry5Ba3Φ*

*Botrytis cinerea* transformed with pTFCM and the six *cry5Ba3Φ*-mutant strains were grown on PDB at 25 °C for 3 days to obtain fungal extracts. Nematodes cultivated on wild-type *B. cinerea* plates (containing approximately 600 nematodes in 50 µl ddH_2_O) were added into each of the extracts. Following 24 and 48 h of contact with the extracts, live or dead nematodes were recorded. Five replicates were used for all the strains studied.

Wild-type and *cry5Ba3Φ*-transgenic *B. cinerea* strains were cultured to collect fungal extracts. The extract of the *cry5Ba3Φ*-transgenic *B. cinerea* strain was supplemented either with 100 µl of 20 mg/ml elastase (Sangon Biotech, Shanghai, China) or with 100 µl sterile ddH_2_O. Forty microliter of PWNs (containing approximately 600 nematodes) was added into each of the extracts (1 ml) mentioned above. Following 48 h of contact with the extracts, live or dead nematodes were recorded. Five replicates were used in all the treatments.

### Statistical analysis

Differences in *cry5Ba3Φ* gene expression and effects on nematode fitness between transformant and wild-type strains were compared by independent samples *t* test. Differences in number of live nematodes and ratio of live:dead nematodes among treatments were performed by one-way analysis of variance (ANOVA). When data were not normally distributed or had no variance homogeneity, the data were rank transformed, log transformed, or were applied for non-parametric Kruskal–Wallis test. The Student–Newman–Keuls (SNK) method or Mann–Whitney U test (with adjusted α-level) was used for pairwise comparisons.

## Results

### Transformation and expression of *cry5Ba3Φ* in *Botrytis cinerea*, the diet fungus of PWN in laboratory

The plasmid pUC57-*cry5Ba3Φ*, prepared by whole-sequence synthesis, was validated by digestion with the restriction enzymes *Xho*I and *Spe*I, resulting in an approximately 2600 bp DNA fragment (*PtrpC*, *cry5Ba3Φ*, and *TtrpC*), which was absent from the gel in case of the plasmid pUC57 (Fig. 1b). The ligated product pTFCM-*cry5Ba3Φ*, combining plasmid pUC57-*cry5Ba3Φ* with plasmid pTFCM, was confirmed by the restriction enzymes *Xho* I and *Sac* I, which cut off an approximately 5600 bp DNA fragment containing expression elements of both *cry5Ba3Φ* and the hygromycin B resistant (*hph*) gene (Fig. 1c). PCR amplification using primers cry-F and cry-R showed that plasmid pTFCM-*cry5Ba3Φ* was successfully carried by *A. tumefaciens* AGL-1 (Fig. 1d).

After co-cultivation of AGL-1 (carrying pTFCM-*cry5Ba3Φ*) and *B. cinerea* conidia on IM with acetosyringone, five *B. cinerea* transformants were picked out from hygromycin B-containing PDA medium. To make sure that the *cry5Ba3Φ*-transgenic fungus received the correct gene for transcription, one of the five fungal transformants was randomly selected for PCR amplification, Southern blotting, and qRT-PCR analyses. Using primers cry-F and cry-R, internal regions of *cry5Ba3Φ* and *hph* were amplified. As shown in Fig. 1e, the expected 1.2 kb fragments were observed at the correct size for *cry5Ba3Φ*-containing *B. cinerea* and plasmid pTFCM-*cry5Ba3Φ*, but not for the wild-type strain of *B. cinerea*. The PCR products were sequenced for confirming the presence of *cry5Ba3Φ* and *hph* genes further. Southern hybridization using the *cry5Ba3Φ* gene fragment as a probe showed that a single copy of T-DNA was integrated into the genome of the *B. cinerea* transformant (Fig. 1f). The qRT-PCR further determined that *cry5Ba3Φ* was expressed only in *B. cinerea* transformant, with an expression quantity approximately six times higher than that of the housekeeping gene *actin* (Fig. 1g).

Assessment of the genetic stability of *cry5Ba3Φ*-containing *B. cinerea* showed that the transformant maintained hygromycin B resistance after being cultured on hygromycin B-free PDA for nine successive generations (5 days for one generation; Additional file 1: Figure S3). The ninth generation was confirmed to contain *cry5Ba3Φ* gene using PCR detection (data not shown).

### Cry5Ba3Φ confers nematicidal activity to *Botrytis cinerea*

**Fig. 2:**
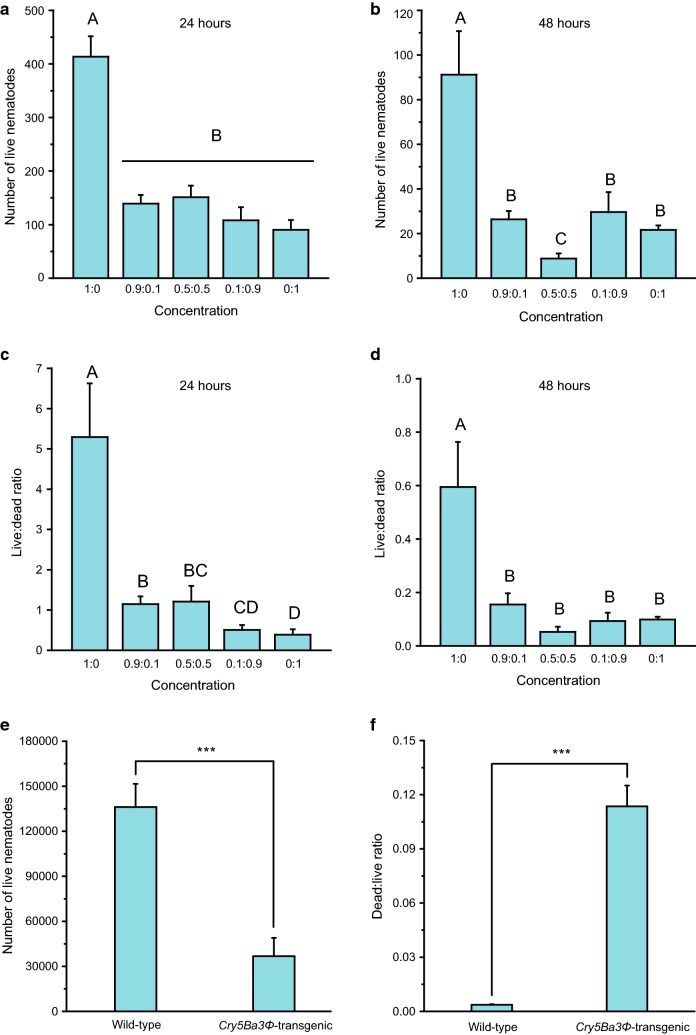
The *cry5Ba3Φ* gene confers strong nematicidal activity to *Botrytis cinerea*. **a** Diet fungus of pinewood nematode (PWN) in the laboratory. Decreased number of live nematodes (**a** 24 h, one-way ANOVA, *F*_4,20_ = 28.227, *P* < 0.0001; **b** 48 h, one-way ANOVA, *F*_4,20_ = 12.319, *P* < 0.0001) and live:dead ratio (**c** 24 h, one-way ANOVA, *F*_4,20_ = 11.735, *P* < 0.0001; **d** 48 h, one-way ANOVA, *F*_4,20_ = 11.168, *P* < 0.0001) with elevated proportion of extracts from *cry5Ba3Φ*-transgenic *Botrytis cinerea*. Fitness loss of PWN population (**e** number of live nematodes, *t* = 5.039, df = 10, *P* < 0.001; **f** dead:live ratio, *t* = − 9.573, df = 5.008, *P* < 0.001) feeding on mycelia of *cry5Ba3Φ*-transgenic *Botrytis cinerea*, after 5 days. *** Indicates *P* < 0.001. Different letters indicate significant differences among treatments (*P* < 0.05)

The *cry5Ba3Φ*-transgenic *B. cinerea* secreted soluble proteins, including the Cry5Ba3Φ protein, which was detected by SDS-PAGE (Fig. 1h). As compared with fungal extracts of the wild-type *B. cinerea*, those supplemented with over 10% extracts of *cry5Ba3Φ*-containing *B. cinerea* caused significantly lower number of live PWNs both after 24 h (Fig. 2a) and 48 h (Fig. 2b). After 24 h, lower live:dead ratio of the nematode was found along with higher proportion of *cry5Ba3Φ*-transgenic *B. cinerea* extract (Fig. 2c). After 48 h, no significant difference in live:dead ratio was found among solutions containing different proportions of transformant extract, but the live:dead ratios of them were all significantly lower than that of the wild-type extract (Fig. 2d). After 5 days, the number of live nematodes feeding on *cry5Ba3Φ*-transgenic *B. cinerea* mycelia was significantly lower than that of live nematodes feeding on the wild-type strain (Fig. 2e). Compared to PWNs on the wild-type *B. cinerea* strain, dead:live ratio of the nematodes was much higher on the transgenic strain (Fig. 2f).

### Differential effects of truncated Cry5Ba3Φ proteins on nematicidal activity

**Fig. 3:**
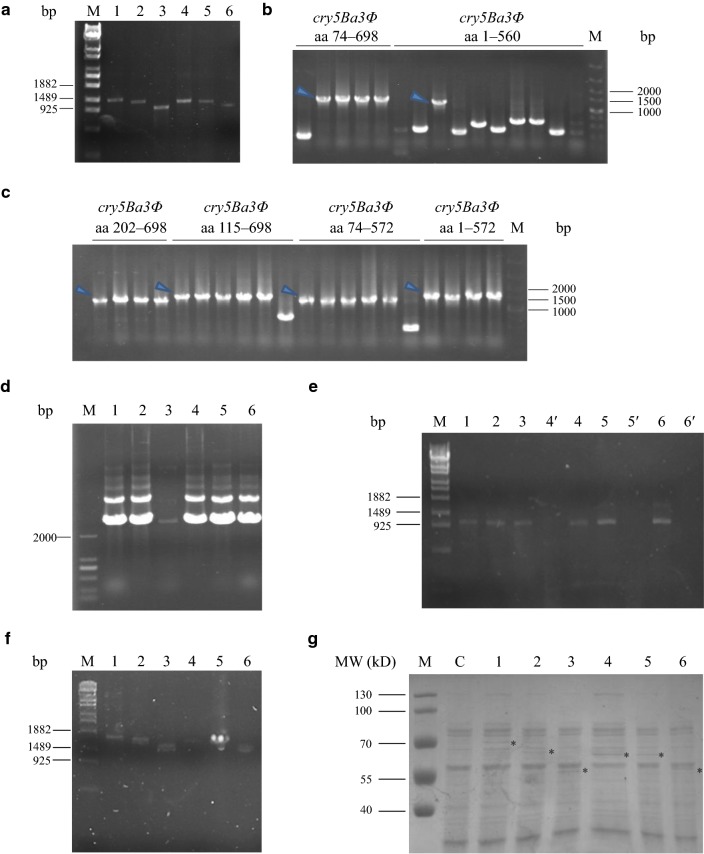
Construction of *Botrytis cinerea* transformants with different lengths of *cry5Ba3Φ*. **a** Six different lengths of *cry5Ba3Φ* gene amplified from the plasmid pTFCM-*cry5Ba3Φ*. 1: *cry5Ba3Φ* aa 74–698; 2: *cry5Ba3Φ* aa 115–698; 3: *cry5Ba3Φ* aa 202–698; 4: *cry5Ba3Φ* aa 1–572; 5: *cry5Ba3Φ* aa 1–560; 6: *cry5Ba3Φ* aa 74–572. **b**, **c** Certification of plasmids pTFCM carrying different lengths of *cry5Ba3Φ* gene. The arrows indicate expected DNA bands amplified from successfully recombined plasmids. **d** Extraction of recombinant plasmids carrying different lengths of *cry5Ba3Φ* gene for AGL-1 transformation. 1: plasmid pTFCM-*cry5Ba3Φ* aa 74–698; 2: pTFCM-*cry5Ba3Φ* aa 115–698; 3: pTFCM-*cry5Ba3Φ* aa 202–698; 4: pTFCM-*cry5Ba3Φ* aa 1–572; 5: pTFCM-*cry5Ba3Φ* aa 1–560; 6: pTFCM-*cry5Ba3Φ* aa 74–572. **e** PCR analysis of AGL-1 transformation confirmed by amplifying the *hph* gene. 1: AGL-1 pTFCM-*cry5Ba3Φ* aa 74–698; 2: AGL-1 pTFCM-*cry5Ba3Φ* aa 115–698; 3: AGL-1 pTFCM-*cry5Ba3Φ* aa 202–698; 4: AGL-1 pTFCM-*cry5Ba3Φ* aa 1–572; 5: AGL-1 pTFCM-*cry5Ba3Φ* aa 1–560; 6: AGL-1 pTFCM-*cry5Ba3Φ* aa 74–572. 4ʹ, 5ʹ, and 6ʹ: failed AGL-1 transformations with corresponding pTFCM plasmids. **f** Identification of *Botrytis cinerea* transformants by amplifying *cry5Ba3Φ* gene of corresponding lengths. **g** SDS-PAGE analysis of soluble proteins produced by *Botrytis cinerea* transformants. Asterisks indicate the protein band of truncated Cry5Ba3Φ. MW: protein molecular weight; M: pre-stained protein marker; C: *Botrytis cinerea* (pTFCM). In (**a**–**f**), M: DNA marker; in **f** and **g**, 1: *Botrytis cinerea* (pTFCM-*cry5Ba3Φ* aa 74–698); 2: *Botrytis cinerea* (pTFCM-*cry5Ba3Φ* aa 115–698); 3: *Botrytis cinerea* (pTFCM-*cry5Ba3Φ* aa 202–698); 4: *Botrytis cinerea* (pTFCM-*cry5Ba3Φ* aa 1–572); 5: *Botrytis cinerea* (pTFCM-*cry5Ba3Φ* aa 1–560); 6: *Botrytis cinerea* (pTFCM-*cry5Ba3Φ* aa 74–572)

Amplified DNA fragments of *cry5Ba3Φ* mutants (Fig. 3a) and pTFCM-TRP vector backbone were assembled and then transformed into *E. coli* XL10-Gold. Putative recombinant plasmids, which showed slower mobility rates than the control on gel, were confirmed by PCR amplification (Fig. 3b, c) and sequencing. The pTFCM plasmids with different lengths of *cry5Ba3Φ* were then extracted for transformation into *A. tumefaciens* AGL-1 (Fig. 3d). Because of the co-occurrence of truncated *cry5Ba3Φ* and *hph* genes, PCR amplification using primers hph-F and hph-R indicated that *cry5Ba3Φ*-truncating genes on the plasmid pTFCM were successfully carried by *A. tumefaciens* AGL-1 (Fig. 3e). Transformant strains of *B. cinerea* containing truncated *cry5Ba3Φ* genes were constructed successfully (Additional file 1: Figure S4), because the *cry5Ba3Φ* gene fragments were amplified by the primers IDF and IDR, which targeted the *cry5Ba3Φ* expression cassette (Fig. 3f). Furthermore, expressed proteins, including truncated Cry5Ba3Φ, were detected from corresponding mutants of *B. cinerea* by SDS-PAGE (Fig. 3g).

**Fig. 4:**
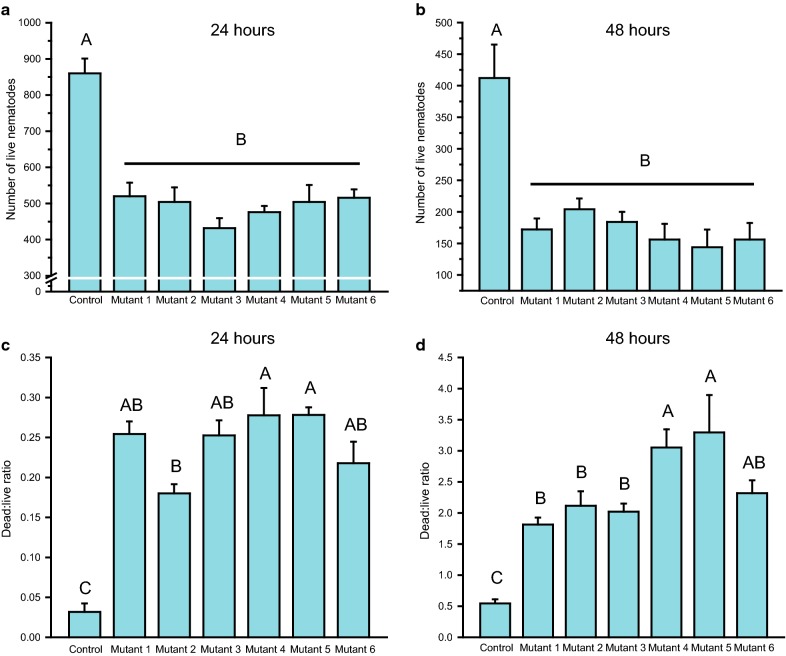
Differential effects of truncated Cry5Ba3Φ proteins on nematicidal activity. **a**, **b** A lower number of live nematodes in the extracts of *cry5Ba3Φ*-mutant *Botrytis cinerea* strains than that in the extracts of *Botrytis cinerea* transformed with pTFCM (control) (24 h, one-way ANOVA, *F*_6,28_ = 16.633, *P* < 0.0001; 48 h, one-way ANOVA, *F*_6,28_ = 10.699, *P* < 0.0001). **c**, **d** Differential dead:live ratio of PWNs among the six *cry5Ba3Φ*-mutant *Botrytis cinerea* strains (24 h, one-way ANOVA, *F*_6,28_ = 22.250, *P* < 0.0001; d: 48 h, one-way ANOVA, *F*_6,28_ = 20.141, *P* < 0.0001). Control: *Botrytis cinerea* (pTFCM); Mutant 1: *Botrytis cinerea* (pTFCM-*cry5Ba3Φ* aa 74–698); Mutant 2: *Botrytis cinerea* (pTFCM-*cry5Ba3Φ* aa 115–698); Mutant 3: *Botrytis cinerea* (pTFCM-*cry5Ba3Φ* aa 202–698); Mutant 4: *Botrytis cinerea* (pTFCM-*cry5Ba3Φ* aa 1–572); Mutant 5: *Botrytis cinerea* (pTFCM-*cry5Ba3Φ* aa 1–560); Mutant 6: *Botrytis cinerea* (pTFCM-*cry5Ba3Φ* aa 74–572). Different letters indicate significant differences among treatments (*P* < 0.05)

**Fig. 5:**
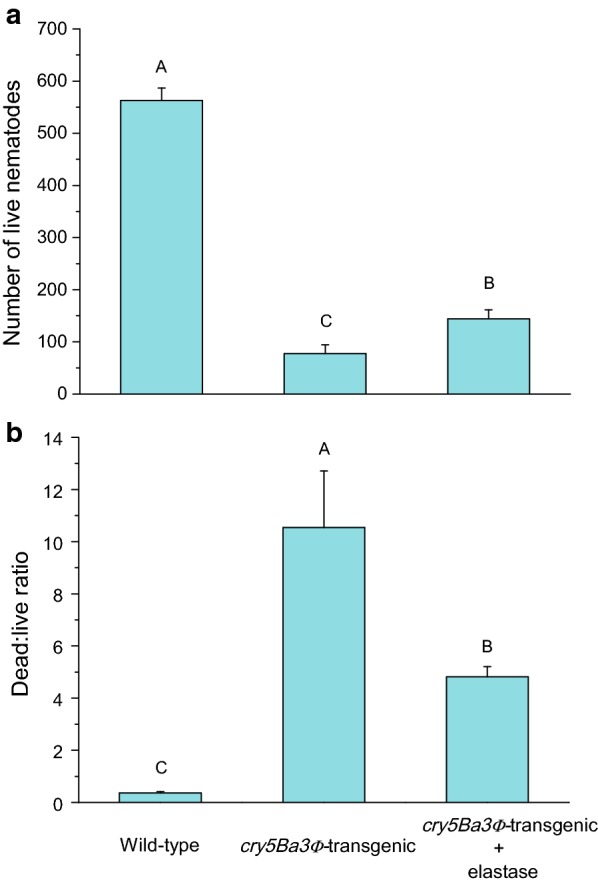
Addition of elastase alleviates Cry5Ba3Φ toxicity against PWN. **a** Number of live nematodes (48 h, one-way ANOVA, *F*_2,12_ = 183.141, *P* < 0.0001). **b** Dead:live ratio of PWN (48 h, Kruskal–Wallis test, χ^2^ = 12.5, df = 2, *P* < 0.01). Different letters indicate significant differences among treatments (*P* < 0.05)

As compared with the treatment using the *B. cinerea* transformed with pTFCM, treatments with all the *cry5Ba3Φ*-truncating mutants resulted in a significantly lower number of live PWNs both after 24 h (Fig. 4a) and 48 h (Fig. 4b). Significant differences were found in dead:live ratio of PWNs among the *B. cinerea* mutant strains. After 24 h, N-terminal deletion of the first 114 amino acids (mutant 2) significantly weakened the toxicity against nematodes, which caused lower dead:live ratio of PWNs than did the C-terminal truncation (mutant 4 and mutant 5; Fig. 4c). After 48 h, N-terminal truncated strains (mutants 1, 2, and 3) showed lower dead:live ratio of PWNs than did the C-terminal truncated strains (mutants 4, 5) (Fig. 4d). Supplementing the extract of the *cry5Ba3Φ*-transgenic strain of *B. cinerea* with elastase significantly alleviated Cry5Ba3Φ toxicity against PWN. After 48 h, as compared with the PWNs in the *cry5Ba3Φ*-transgenic strain extract without elastase, the nematodes in the extract containing elastase had higher number of individuals in the population (Fig. 5a) and lower dead:live ratio (Fig. 5b).

## Discussion

Bt crystal proteins are toxic to a variety of nematodes, particularly those from Cry5 subfamily [36–38]. Multiple free-living nematode species exhibited susceptibilities to Cry5B, Cry14A, and Cry21A [38]. Cry5A, Cry5B, and Cry13 proteins were identified with significant inhibition against the free-living larval stages of nematode parasites of livestock [39]. Cry5B was isolated from supernatant of *B. thuringiensis* AB88 culture [40]. This protein was further found to be highly active in vivo against the human nematode parasite *Ancylostoma ceylanicum* [41] and was shown to have excellent potential as a control agent of the root-knot nematode *Meloidogyne incognita* [26], suggesting its wide-spectrum virulence on various parasitic nematodes. Phylogenetic analysis indicated high similarity of amino acid sequences between Cry5Ba3 and Cry5B (Additional file 1: Figure S1), supporting the toxicity of Cry5Ba3 or Cry5Ba3Φ protein against the PWNs (Fig. 2). Other toxins, belonging to the Cry5 subfamily, such as Cry5Ca1, Cry5Da1, and Cry14 were recently found to act on PPNs, such as *M. incognita* and *M. javanica* [42, 43]. The nematicidal *cry5* subfamily genes, however, appeared to occur in low frequency in natural environments [44]. This finding was consistent with our previous study, which showed that strikingly low frequency of *cry* genes in this subfamily can be detected in the soil samples collected [33].

Insecticidal Cry proteins generally contain three domains, and the function of each domain has been studied. In previous studies, researchers argued that domain I of Cry proteins was involved in the perforation of the intestinal tract during the insecticidal process [45, 46]. Domain II determined the insecticidal specificity of the Cry proteins [47, 48], binding to the gut cadherin receptors of target insects [47, 49–51]. Domain III appeared to prevent Cry proteins from being excessively degraded by proteases in the intestine of insects [52] and to modulate the permeability of gut epithelial cell channels [47]. The nematicidal Cry5B has been crystallized for structure determination. It showed a familiar three-domain arrangement seen in insecticidal Cry proteins, but with a more structurally divergent domain II, which was implicated in interaction with glycolipid receptors of nematodes [53, 54].

Considering the high similarity of amino acid sequences between Cry5Ba3 and Cry5B, the putative domain II of Cry5Ba3Φ was retained without any excision, whereas domain I (in endotoxin-N) and III (in endotoxin-C) were designed to be broken down to detect weakening in nematicidal activity after the protein expression in *B. cinerea*. As compared with the wild-type *B. cinerea* strain, extracts from all the *cry5Ba3Φ*-mutant strains displayed stronger toxicities against PWNs (Fig. 4a, b). In contrast, differential virulence was found among the mutant strains of *B. cinerea*, particularly for their effect on dead:live ratio of the nematodes (Fig. 4c, d). The virulence of N-terminal mutants to nematodes was significantly impaired, while the mutant strains expressing C-terminal truncated Cry5Ba3Φ proteins had greater toxicities than other mutant types. This implied that N-terminal portion (domain I) was of greater significance for the toxicity of Cry5Ba3Φ protein than was the C-terminal portion (domain III). For the closely related protein Cry5B, cleavage by elastase, which disintegrated the N-terminal portion of this protein, yielded two residues of 112–170 and 173–698 amino acids [54]. In the present study, supplementing the extract of the *cry5Ba3Φ*-transgenic strain of *B. cinerea* with elastase caused a significant decrease in its toxicity against PWN (Fig. 5), which further supported the crucial role of domain I of Cry5Ba3Φ in nematicidal ability. In the future, variations in the glycolipid-binding activities of truncated Cry5Ba3Φ proteins and their structures should be determined. Interesting, the loss of segments anterior to endotoxin-N also affected Cry5Ba3Φ nematicidal activity, which needs to be further demonstrated by experiments.

Mutagenesis using ATMT has been widely applied into filamentous fungi, including *Aspergillus awamori*, *Penicillium digitatum*, and *Umbilicaria muehlenbergii* [55–58]. In this study, ATMT was used to generate insertional mutation in the fungus, *B. cinerea*. The strong nematicidal *cry5Ba3Φ* gene was successfully carried and expressed by this diet fungus of PWNs. PCRs and Southern hybridizations demonstrated that *cry5Ba3Φ* gene was integrated into *B. cinerea* as a single copy, with expression level approximately six times higher than that of the housekeeping *actin* gene. These studies and our previous research might be among the first comprehensive attempts of searching for a novel Bt-based control strategy against PWN. In this process, *B. thuringiensis* strains were isolated from soils, a Cry protein was identified to have efficient nematicidal effects on PWN, and the *cry* gene was transformed into *B. cinerea*. The *cry*-transformed *B. cinerea* was found to be toxic to PWN, and finally, various *cry* mutant strains of *B. cinerea* were constructed to characterize the structure–function relationship of the Cry protein.

*Botrytis cinerea* may be applied as a prospective attractant for PWN in the field, albeit in usual as a diet fungus for laboratory PWN population. *B. cinerea*, *Pestalotia*, and Microzyme have been compared in their capabilities to attract PWN. *Botrytis cinerea* was most attractive to PWN among these fungi, possibly through secretion of extracellular active substances [59]. This implied that *B. cinerea* is a suitable expression receptor for nematicidal *cry* genes, which could be developed as a “sweet toxin” biocontrol agent for PWNs. Recently, the recombination of symbiotic microbes of pests for producing toxic molecules has attracted attention, and some of these achieved efficient control. For instance, with the purpose of controlling human malaria parasite *Plasmodium falciparum*, symbiotic bacteria of its vector (*Anopheles* mosquito) were genetically engineered for secretion of anti-*Plasmodium* effector proteins to interfere with the development of *P. falciparum* in mosquitoes [60, 61]. Therefore, the platform we constructed using *B. cinerea* would inspire explorations of fungal mutualists, naturally associated with the PWN-vector *Monochamus* complex, which are genetically engineered to secrete nematicidal Cry proteins. A recent study reported that a native fungal symbiont, *Sporothrix* sp. 1, was dominant in the sites where tree infestation was higher, and its presence significantly improved the fitness of both PWN and the vector *M. alternatus* [32]. This finding may provide an opportunity for further genetic engineering to produce recombinant *Sporothrix* sp. 1 carrying *cry* genes in the future. Symbioses affect the pest status of many groups of insects and nematodes, signifying a wide prospect for the use of genetically modified symbiotic microbes as powerful tools for combating forest and agricultural animal pests.

## Conclusions

The data presented in this study demonstrate that nematicidal Cry5Ba3 found in a soil *B. thuringiensis* strain can be successfully expressed in *B. cinerea*, a diet fungus of PWN in the laboratory, via ATMT technology. The mortality of the nematodes exposed to extracts from *cry5Ba3Φ*-containing *B. cinerea* is comparable with that in the presence of purified Cry5Ba3, as previously reported. In addition, our results indicate that impairing Cry5Ba3Φ at different loci result in distinct levels of effects on its toxicity against PWN. These findings will not only inspire explorations into Bt-transgenic fungal mutualists of PWN, but also help in establishing a platform suitable for characterizing the structure–function relationships of various candidate Cry proteins against this nematode pest.

## Additional file

**Additional file 1.** This file includes: **Methods. Figure S1.** Phylogenetic analysis of Cry5Ba3 with other homologous cry5 subfamily proteins. **Figure S2.** Codon modification of *cry5Ba3* to *cry5Ba3Φ*. **Figure S3.** Fungal colony morphologies. **Figure S4.** Fungal colony morphologies of *Botrytis cinerea* transformants with different lengths of *cry5Ba3Φ*.

## Abbreviations

PWN: pinewood nematode
Bt: *Bacillus thuringiensis*
ATMT: *Agrobacterium tumefaciens*-mediated transformation
PPN: plant-parasitic nematodes
Cry: crystal
PDA: potato dextrose agar
LB: Luria–Bertani
IM: induction medium
AS: acetosyringone
OD: optical density
CTAB: cetyltrimethylammonium bromide
PCR: polymerase chain reaction
EDTA: ethylenediaminetetraacetic acid
SSC: saline sodium citrate
SDS: sodium dodecyl sulfate
NCBI: National Center for Biotechnology Information
CD-search: conserved domain search
SDS-PAGE: sodium dodecyl sulphate polyacrylamide gel electrophoresis
ANOVA: analysis of variance
SNK: Student–Newman–Keuls
