# Supplementary material for: Suppressing a plant-parasitic nematode with fungivorous behavior by fungal transformation of a Bt cry gene
Source: Microb Cell Fact. 2018 Jul 23;17:116. doi: 10.1186/s12934-018-0960-5 (PMC6055344; doi:10.1186/s12934-018-0960-5)

Additional file 1:

Suppressing a plant-parasitic nematode with fungivorous behavior by fungal transformation of a Bt *cry* gene

Chihang CHENG[1, 2#], Jialing QIN[1, 3#], Choufei WU[1, 2], Mengying LEI[4], Yongjun WANG[1, 3*], Liqin ZHANG[1, 2*]

[1] Collaborative Innovation Center of Zhejiang Green Pesticide, School of Forestry and Biotechnology, Zhejiang A&F University, Hangzhou, 311300, China

[2] School of Life sciences, Huzhou University, Huzhou, 313000, China

[3] State Key Laboratory of Subtropical Silviculture, School of Forestry and Biotechnology, Zhejiang A&F University, Hangzhou, 311300, China

[4] Guangdong Eco-Engineering Polytechnic, Guangdong, 510520, China

[#] These authors contributed equally to this paper.

[*] *To whom for Correspondence*: Yongjun WANG, wangyj@zafu.edu.cn; Liqin ZHANG, zlqwz@zjhu.edu.cn.

Methods

PCR amplifying DNA fragments of *cry5Ba3Φ* gene with different lengths

Primers prepared for amplification of *cry5Ba3Φ* fragments were listed following:

cry5Ba3Φ aa 74–698 (1,875bp):

74–698F:

TTCTACCCAAGCATCCAAGATATGAAGGCTTCCATCTCCCTCATC

74–698R:

TCCCGGTCGGCATCTACTGATTTATTGAATCTTTGGAACGAATTCGA

cry5Ba3Φ aa 115–698 (1,758bp):

115–698F:

TTCTACCCAAGCATCCAAGATATGCAACTCTTCAACGCTATCATGG

115–698R:

TCCCGGTCGGCATCTACTGATTTATTGAATCTTTGGAACGAATTCGA

cry5Ba3Φ aa 202–698 (1,497bp):

202–698F:

TTCTACCCAAGCATCCAAGATATGTCCCAATTCACCCAACATCTC

202–698R:

TCCCGGTCGGCATCTACTGATTTATTGAATCTTTGGAACGAATTCGA

cry5Ba3Φ aa 1–572 (1,719bp):

1–572F:

TTCTACCCAAGCATCCAAGATATGGCTACCATCAACGAACTCTACC

1–572R:

TCCCGGTCGGCATCTACTGATTTATTCGGAACCGTACTTTTCGAAT

cry5Ba3Φ aa 1–560 (1,683bp):

1–560F:

TTCTACCCAAGCATCCAAGATATGGCTACCATCAACGAACTCTACC

1–560R:

TCCCGGTCGGCATCTACTGATTTATTGGGTGAGTGGGAGCTTGG

cry5Ba3Φ aa 74–572 (1,503bp):

74–572F:

TTCTACCCAAGCATCCAAGATATGAAGGCTTCCATCTCCCTCATC

74–572R:

TCCCGGTCGGCATCTACTGATTTATTCGGAACCGTACTTTTCGAAT

PCR reaction consisted of 0.2 μg of pTFCM-*cry5Ba3Φ* template, 2.5 U Pfu DNA polymerase, 5 μl 10× PCR buffer, 4 μl 12.5× dNTPs and 5 μl of 10 μmol/L each primer, adding ddH_2O to 50 μl. The cycling conditions were as follows: an initial denaturation of 2 min at 94 °C, followed by 25 cycles of 30 s for denaturation at 94 °C, 30 s for annealing at 55 °C, and 1 min for polymerization at 72 °C, with a final extension of 72 °C for 7 min.

PCR amplifying pTFCM-TRP vector backbone

pTFCM-phiF/-phiR was used as primers (pTFCM-phiF: 5′-TACCTATTCTACCCAAGCATCCAAGATATCAGTAGATGCCGACCGGGA-3′; pTFCM-phiR: 5′-TTGGATGCTTGGGTAGAATAGGT-3′) to amplify pTFCM-TRP vector backbone, which resulted in an approximately 11 kb product of pTFCM including *trpC* promoter and terminator. PCR reaction consisted of 0.2 μg of pTFCM-*cry5Ba3Φ* template, 2.5 U Pfu DNA polymerase, 5 μl 10× PCR buffer, 4 μl 12.5× dNTPs and 5 μl of 10 μmol/L each primer, adding ddH_2O to 50 μl. The cycling conditions were as follows: an initial denaturation of 2 min at 94 °C, followed by 25 cycles of 30 s for denaturation at 94 °C, 30 s for annealing at 55 °C, and 5 min for polymerization at 72 °C, with a final extension of 72 °C for 7 min.

PCR Certification of *B. cinerea* with truncated *cry5Ba3Φ*

Genomic DNAs of the 6 *B. cinerea* transformant strains were extracted for PCR amplification to certify the presence of truncated *cry5Ba3Φ* genes, using primers IDF (5′- ACTAGTCATTGCAGATGAGCTG-3′) and IDR (5′-ACTAGTCATTGCAGATGAGCTGTATCTGGA-3′). PCR reaction consisted of 0.2 µg of DNA template, 1 U Pfu DNA polymerase, 1 µl 10× PCR buffer, 0.8 µl 12.5× dNTPs and 1 µl of 10 µmol/L each primer, adding ddH_2O to 10 µl. The cycling conditions were as follows: an initial denaturation of 2 min at 94 °C, followed by 25 cycles of 30 s for denaturation at 94 °C, 30 s for annealing at 55 °C, and 1 min for polymerization at 72 °C, with a final extension of 72 °C for 7 min.

Figures

Figure S1. Phylogenetic analysis of Cry5Ba3 with other homologous cry5 subfamily proteins. DNA sequences were translated into amino acid data and were outputted for tree construction using the neighbor-joining (NJ) method. Cyt1Aa1 was set as the outgroup to root the phylogeny. The bootstrap test was used to value the relative support for each node with 1000 replicates. GenBank accession numbers were presented after protein names.

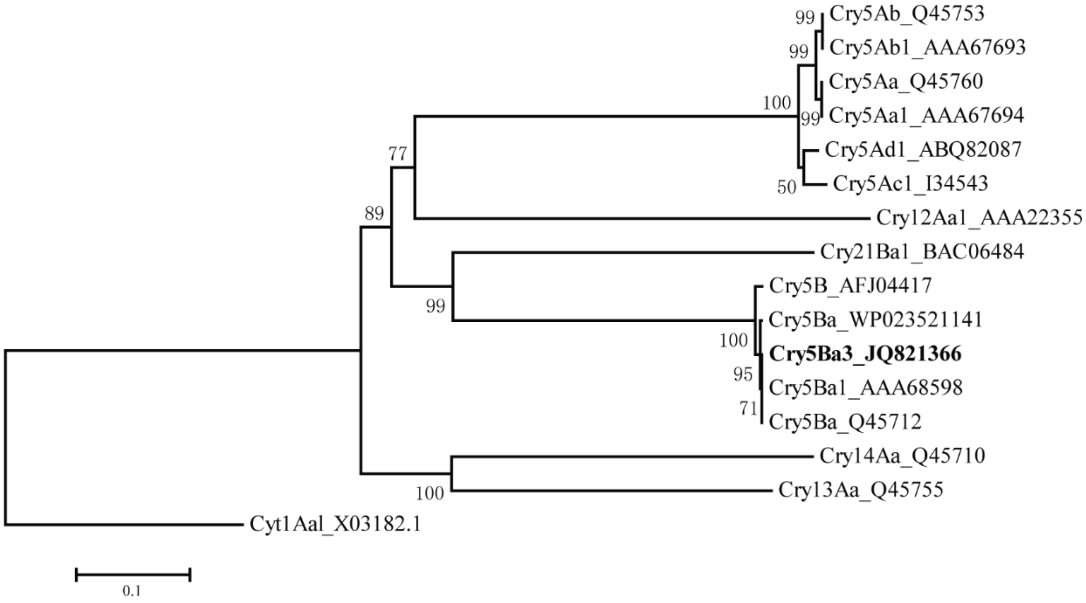

Figure S2. Codon modification of *cry5Ba3* to *cry5Ba3Φ*.

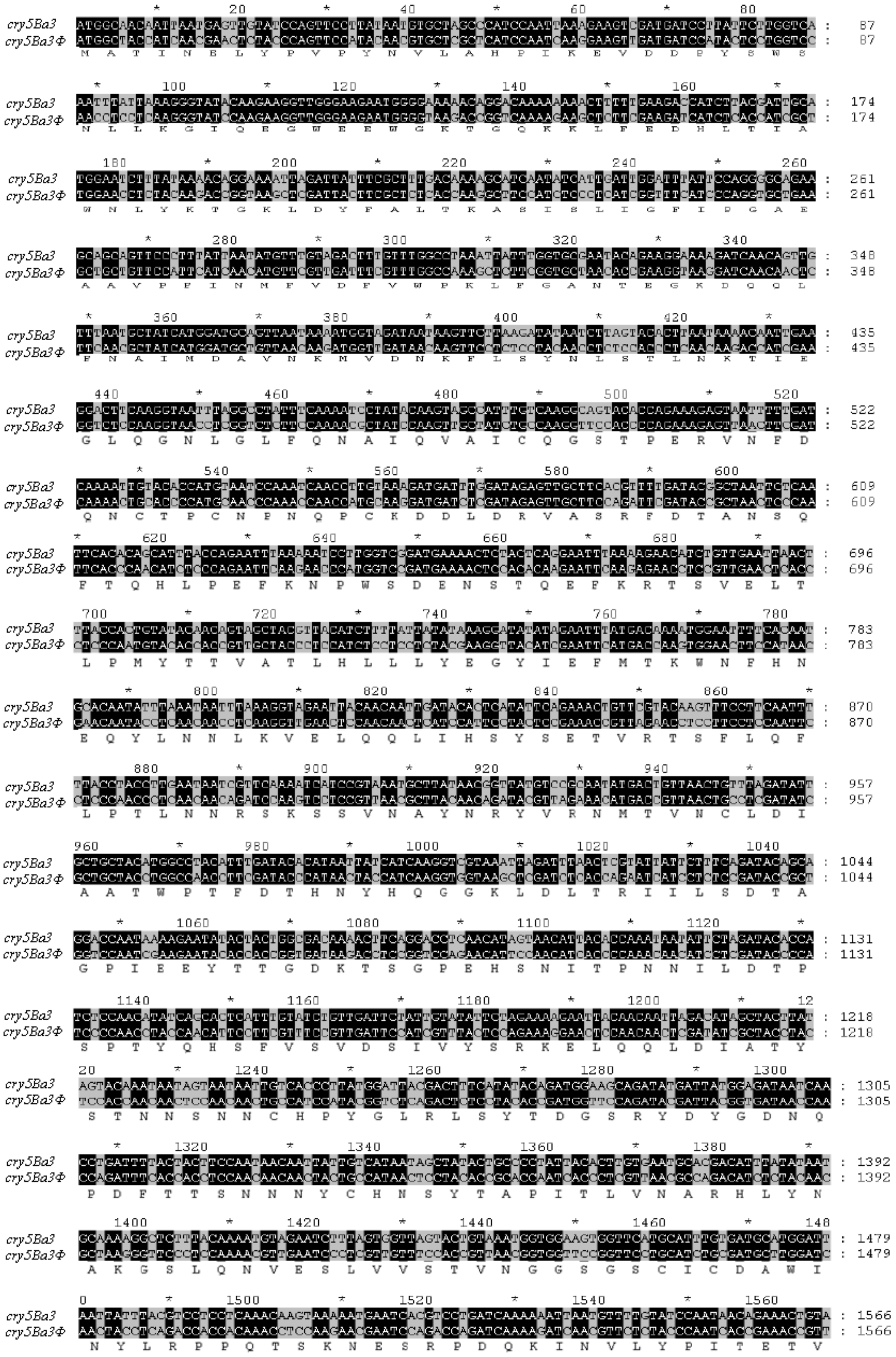

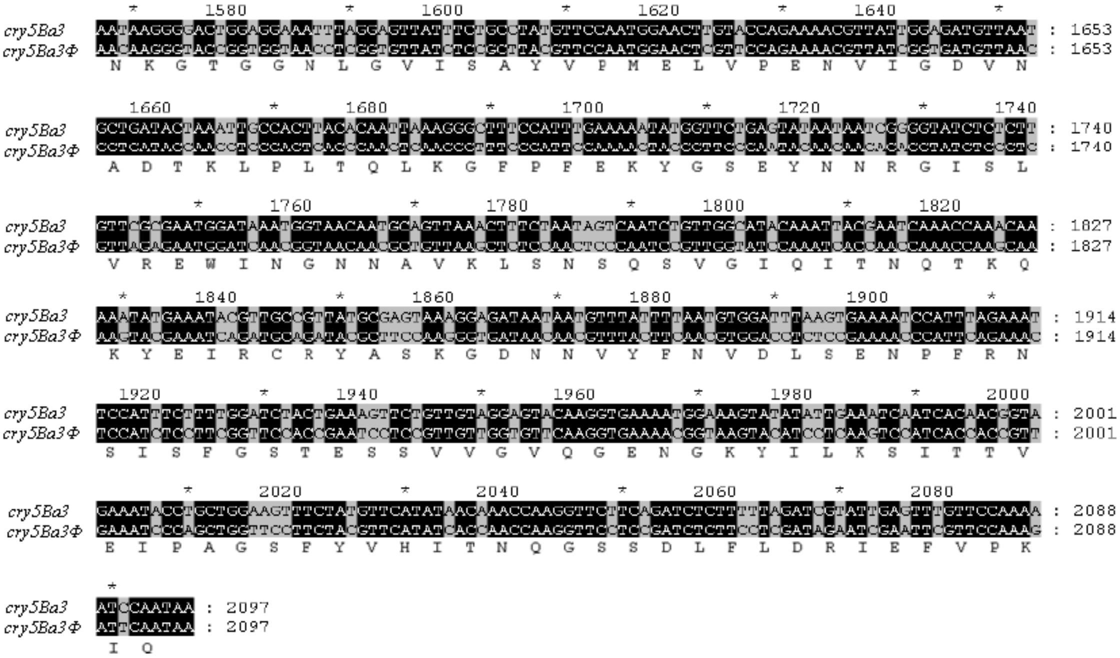
cry5Ba3
cry5Ba3Φ
1580 1600 1620 1640
: 1653
N K G T G G N L G V I S A Y V P M E L V P E N V I G D V N
1660 1680 1700 1720 1740
: 1740
A D T K L P L T Q L K G F P F E K Y G S E Y N N R G I S L
1760 1780 1800 1820
: 1827
V R E W I N G N N A V K L S N S Q S V G I Q I T N Q T K Q
1840 1860 1880 1900
: 1914
K Y E I R C R Y A S K G D N N V Y F N V D L S E N P F R N
1920 1940 1960 1980 2000
: 2001
S I S F G S T E S S V V G V Q G E N G K Y I L K S I T T V
2020 2040 2060 2080
: 2088
E I P A G S F Y V H I T N Q G S S D L F L D R I E F V P K
: 2097
I Q

Figure S3. Fungal colony morphologies. (a) Wild-type *Botrytis cinerea*. (b) *cry5Ba3Φ-transgenic Botrytis cinerea*. (c) The ninth-generation strain of *cry5Ba3Φ-transgenic Botrytis cinerea*.

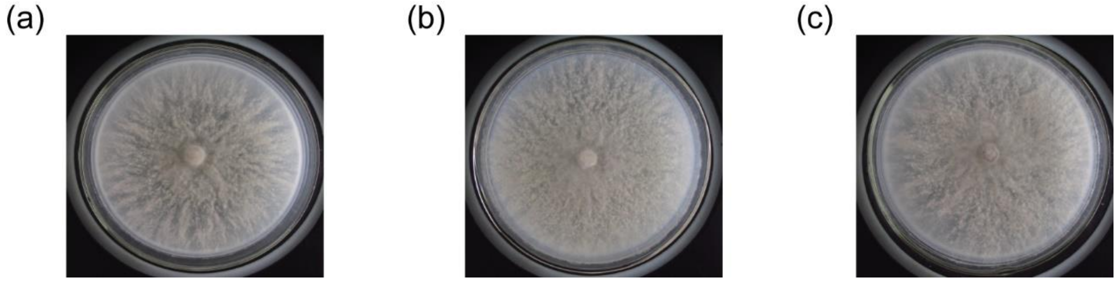

Figure S4. Fungal colony morphologies of *Botrytis cinerea* transformants with different lengths of *cry5Ba3Φ*. (a) *Botrytis cinerea* (pTFCM-*cry5Ba3Φ* aa 74–698). (b) *Botrytis cinerea* (pTFCM-*cry5Ba3Φ* aa 115–698). (c) *Botrytis cinerea* (pTFCM-*cry5Ba3Φ* aa 202–698). (d) *Botrytis cinerea* (pTFCM-*cry5Ba3Φ* aa 1–572). (e) *Botrytis cinerea* (pTFCM-*cry5Ba3Φ* aa 1–560). (f) *Botrytis cinerea* (pTFCM-*cry5Ba3Φ* aa 74–572).

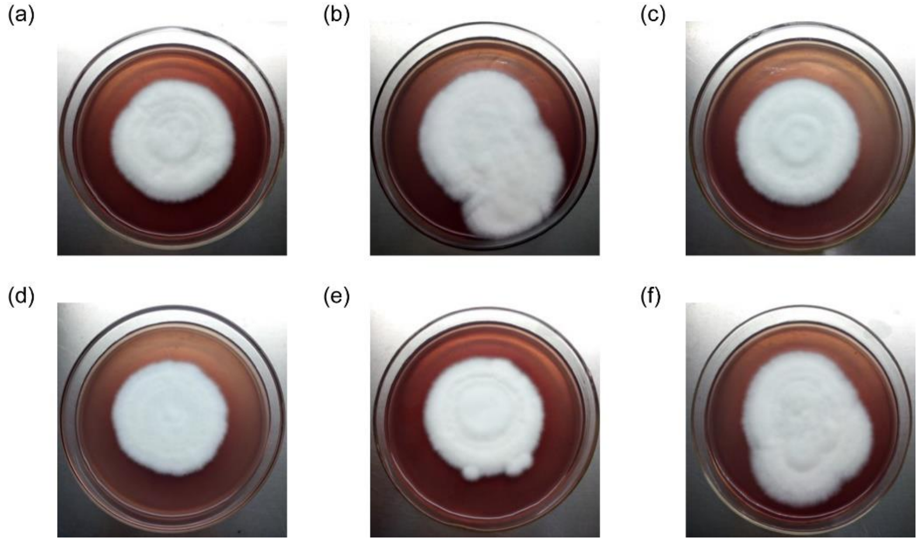

Supplement: Supplementary file 1 — Additional file 1. This file includes: Methods. Figure S1. Phylogenetic analysis of Cry5Ba3 with other homologous cry5 subfamily proteins. Figure S2. Codon modification of cry5Ba3 to cry5Ba3Φ. Figure S3. Fungal colony morphologies. Figure S4. Fungal colony morphologies of Botrytis cinerea transformants with different lengths of cry5Ba3Φ. [file 12934_2018_960_MOESM1_ESM.pdf]
